# Correlations of left ventricular systolic function indices with aortic root systolic excursion (ARSE): A cross-sectional echocardiographic study

**DOI:** 10.1371/journal.pone.0206199

**Published:** 2018-11-06

**Authors:** Ahmadou M. Jingi, Ba Hamadou, Jean Jacques Noubiap, Liliane Mfeukeu-Kuate, Jerome Boombhi, Chris Nadege Nganou, Narcisse Assene Ateba, Aude Laetitia Ndoadoumgue, Ulrich Flore Nyaga, Alain Menanga, Samuel Kingue

**Affiliations:** 1 Department of Medicine and Specialties, Faculty of Medicine and Biomedical Sciences, University of Yaoundé 1, Yaoundé, Cameroon; 2 Cardiology Unit, Yaoundé Central Hospital, Yaoundé, Cameroon; 3 Department of Medicine, University of Cape Town and Groote Schuur Hospital, Cape Town, South Africa; 4 Cardiology Unit, Yaoundé General Hospital, Yaoundé, Cameroon; Wayne State University, UNITED STATES

## Abstract

**Background:**

Heart failure (HF), is a major public health issue globally. Echocardiography is cost–effective in the diagnosis in expert hands. This study was conducted to estimate the usefulness of Aortic Root Systolic Excursion (ARSE) as a simple and accurate measure to estimate Left Ventricular (LV) function.

**Methods:**

This was a cross-sectional echocardiographic study among adults aged ≥ 18 years, with or without heart failure, in sinus rhythm, and with no LV out–let obstruction. We studied the correlations of ARSE with some selected indices of LV Systolic Functions. We determined optimal cut–offs of ARSE in detecting LV dysfunction. We generated a simple regression equation to best estimate LV ejection fraction according to the modified Simpson method.

**Results:**

Overall 213 echocardiograms were included from 106 males (49.8%), with mean age of the participants being 52.4 (SD: 18.3) years. The rate of LV systolic dysfunction was highest with Teicholz method (17.4%) and lowest with MAPSE method (5.2%). ARSE correlated with the LV functions. This was highest for the Simpson method (r = 0.619, p<0.001), and lowest for the ITV method (r = 0.319, p<0.001). Optimal cut-offs to detect LV systolic dysfunction was ≈ 6.6 mm. For an LV ejection fraction < 55%, the sensitivity was 82.9%, and the specificity was 97.2%, with an AUROC of 91.6%.The logarithmic regression equation was best in predicting LV ejection fraction (AUC: 60.2%), followed by the power model (AUC: 56.7%), and the linear model (AUC: 53.6%).

**Conclusion:**

ARSE correlated well with LV systolic function. The cut–off ≤ 6.5 mm suggest LV systolic dysfunction. LV Ejection Fraction was best estimated with the generic equation: LVEF (%) = 29 x In [ARSE].

## Introduction

Heart failure (HF), which corroborates with high rates of hypertension and other cardiovascular risk factors is becoming an established public health issue world-wide. Precise diagnosis therefore is necessary for efficient management and follow-up of patients with heart failure [1]. The increasing rates of hypertension worldwide, has the greatest impact in low-income settings [2–4]. This is coupled with low rates of awareness, detection, and limited access to investigation tests and appropriate anti-hypertensive treatment [5–7]. The multitude of patients with undiagnosed and untreated hypertension will eventually develop heart failure that will require adequate investigations and treatment. This high burden of hypertension and hypertensive heart disease in low income settings is followed by heart failure due to the idiopathic dilated cardiomyopathies and rheumatic heart disease [2]. Unfortunately, most of these patients with heart failure do not have access to skilled cardiologist care. There are many techniques of investigations in the setting of heart failure. Transthoracic echocardiography is a user friendly, cost effective, and non-invasive investigation technique that can be used at the bedside of patients [8]. There are many echocardiographic indices to evaluate left ventricular function [9,10]. The results however depend on the skills of the operator, and the performance of the echocardiograph used. These two criteria are not frequently met in daily clinical practice in resource limited settings. Ünlüer et al [11] studied the relationship of aortic root excursion with left ventricular ejection fraction (LVEF) in the emergency setting. Their findings however could not detect LVEF lower than twenty percent despite a good correlation. Tandon et al [12] studied the correlation of aortic root motion with left ventricular diastolic function. Their procedures were however not suitable for the busy or less skilled physician in low- income settings, who are often faced with patients with heart failure. Also, most of them might lack the basic echocardiography skills needed for the diagnosis and differentiating between heart failure due to low or preserved ejection fractions. With the hypothesis that aortic root motion during left ventricular systole correlated with left ventricular systolic ejection fraction as reported by Ünlüer et al [11], the amplitude of displacement can be a surrogate of left ventricular radial and longitudinal functions. These methods however need to be improved, and their application in low-income settings like ours need to be verified or studied further. There is thus a need for simpler measurements like the amplitude of aortic root motion in systole. We carried out this cross-sectional echocardiographic study with the aim of deriving a simple, rapid and more accurate estimate of ventricular function. This can be particularly useful as an initial screening technique for the under-skilled physician in areas where there is no cardiologist.

## Methods

### Ethical consideration

This work was approved by the institutional review board of the Faculty of Medicine and Biomedical Sciences, University of Yaounde 1. Local authorisation was obtained from the hospital administration. We carried out this work in accordance with the declarations of Helsinki [12].

### Study design and study setting

This was a prospective cross-sectional and analytical study. We report this work according to the Standards for Reporting Studies on Diagnostic Accuracy (STARD) checklist [13]. We carried out this study in the echocardiography laboratory of the Yaoundé Central Hospital—Cameroon, between January and July 2016. This centre is staffed with heart specialists (5 cardiologists), and is equiped with a state-of-the-art ultrasound echocardiograph (Philips). The population of Cameroon is estimated at 23 million inhabitants, of which 64 are cardiologists distributed in two cities of two neighboring regions.

### Study population

Consenting adult participants of both sexes, aged ≥ 18 years seen in the echocardiography laboratories of the teaching hospitals were potentially eligible for the study. Participants were either outpatients or stable inpatients in whom cardiac ultrasound was requested. Oral consent was obtained for each participant or a relative. Non included were; non-adult participants aged < 18 years. Irregular rhythm as in atrial fibrillation might have markedly fluctuationg functions. Left ventricular outflow obstruction such as in obstructive cardiomyopathy or significant valvular pathology like aortic valve stenosis hindering blood flow. Severe mitral valve stenosis with markedly dilated left atrium preventing aortic root descend. Pregnant women with modified cardiac hemodynamics. Intubated patients with modified cardiac hemodynamics. Chronic thrombo-embolism that will reduce aortic root excursion [14]. Large pericardial or pleural effusions from any cause with hemodynamic effects. Chest deformity that will hinder optimal exploration. Poor acoustic window that will hinder optimal exploration. Non-consent of the patient or participant.

### Sample size and power

To achieve significant correlations between aortic root indices and left ventricular functions with set value of r = 0.6, a type 1 error of 0.05 and a power of 80% to detect significant trends, and with an expected prevalence of left ventricular dysfunction of 50% on echocardiograms, a minimum size of 28 participants (14 participants with normal Left Ventricular function and 14 participants with Left Ventricular dysfunction) was required. We enrolled more than this number due to the high volume of cardiac ultrasounds performed in this laboartory so as to achieve a power of over 90%, with an acceptable alpha error of 0.05 or less.

### Procedure and measurements

With all the participants in the left lateral position, the amplitude of the aortic root movement (mm) during systole in TM mode (S1 Fig) was measured from the far wall (average of 3 measures) in the long parasternal window view after zooming of the aortic root. Left Ventricuar (LV) systolic function indices (Ejection fraction using the biplane Simpson and Teicholz methods, Fractional shortening, Mitral Annular Plane Systolic Excursion, Velocity Time Integral) of the LV outflow tract were measured (average of 3 measures) in the apical chambers views. All echocardiograms and measurements were performed by an experienced cardiologist (BH) assisted by the principal investigator (AMJ). Our working definitions or cut-offs are based on the guidelines of the American Society of Echocardiography [9]. Low Left Ventricular Ejection Fraction (LVEF) was defined as LVEF < 55%. Low Mitral Annular Plane Systolic Excursion (MAPSE) was defined as MAPSE < 10mm. Low Aortic Velocity Time Integral (VTI) was defined as VTI < 16 cm. Low cardiac output was retained when it was < 2.5 l/min.

### Statistical analysis

The data were analyzed with the Statistical Package for Social Science (SPSS) version 16.0 for Windows (SPSS, Chicago, Illinois, USA). The left ventricular systolic function indices were plotted against the mean values of the amplitude of the aortic root movement in Systole (ARSE). Regression equation models were derived from these scatter plots. The model with the best fit (best coefficient of determination: R^2^) was considerd in predicting LV systolic function (LVEF) from the amplitude of ARSE. Receiver Operator Characteristic (ROC) curves were plotted, from which optimum thresholds of ARSE that give the best sensitivity-specificity pair (Youden index = Sensitivity + Specificity—1) were derived. The predictive values and likelihood ratios of the derived thresholds were studied (using MedCal online calculator). We used Bland and Altman plots to study the agreement between LVEF calculated by the biplane Simpson method, and LVEF estimated with the ARSE amplitude generic equation. We then performed regression analysis to look for proportional bias. The optimal threshold of the amplitude of ARSE to predict low LVEF (<55%) was externally validated in an independent sample of participants. We presented continuous variables as means with the standard deviation (SD), and proportions with the 95% confidence intervals (95% CI) for discrete variables. For observed differences or degree of association between variables, a p value < 0.05 was considered statistically significant.

## Results

### Participants

**Fig 1:**
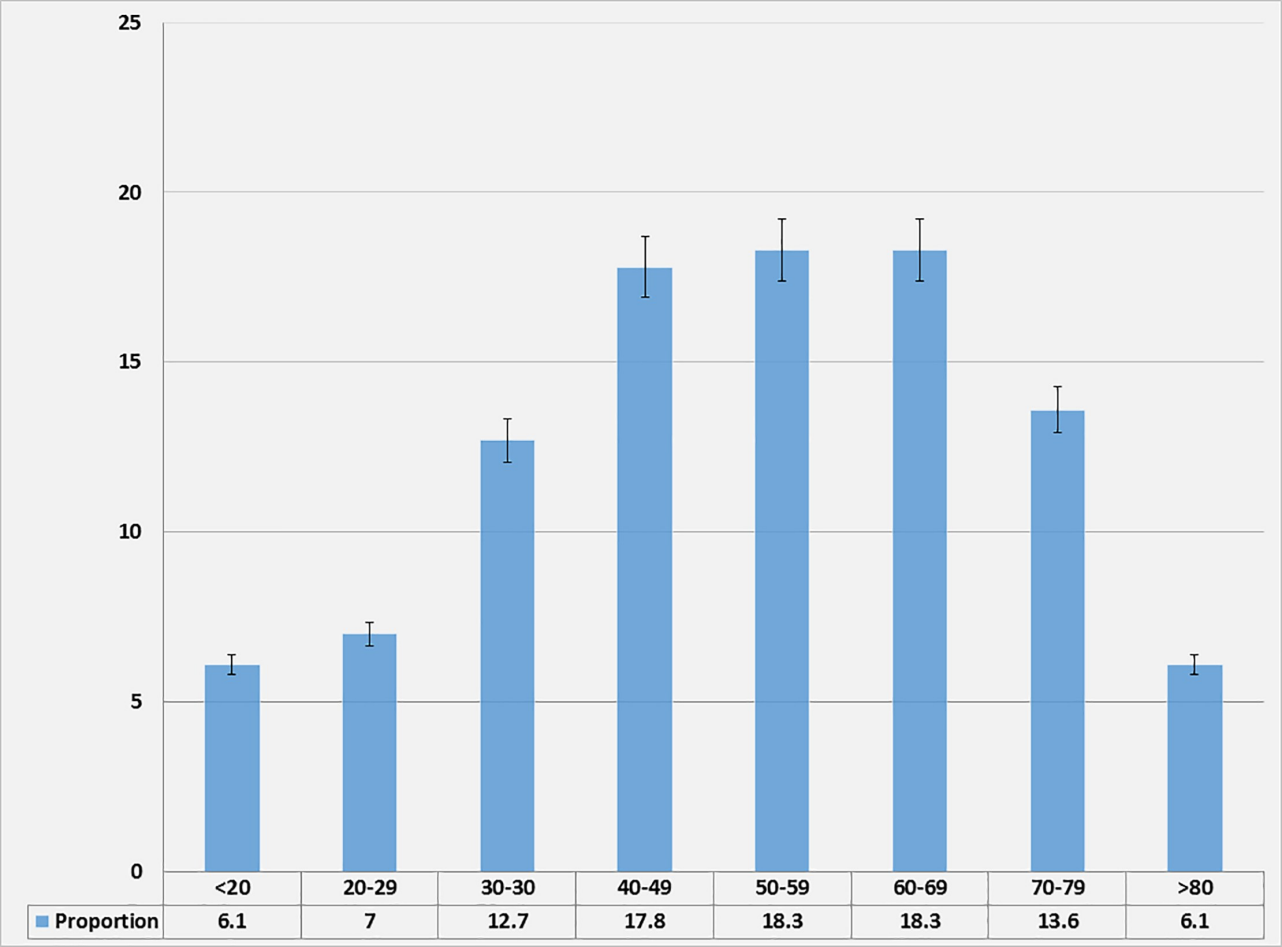
Age distribution of the study population.

A total of 213 echocardiograms of participants were included in this study. There were 106 males (49.8%, [95% CI: 43.1–56.5]) and 107 females (50.2%, [95% CI: 43.5–56.9]), with an overall mean age of 52.4 (SD: 18.3) years (95% CI: 49.9–54.9). Their ages ranged from 18 to 90 years, and most of them were in the 40 to 60 years age group (Fig 1). There was no trend in the distribution of low ejection fraction according to age group. A second independent sample of 32 echocardiograms was used to externally validate our findings.

### Descriptive and outcome data

Clinical and echocardiographic characteristics according to LV Ejection Fraction (LVEF < 55% versus LVEF ≥ 55%) assessed by the modified Simpson method is shown in Table 1. age, gender distribution, Body Mass Index (BMI), and wall thickness were similar between groups. Patients with low LVEF had significantly dilated LV and LV mass.

**Table 1:** Clinical and echocardiographic characteristics of the study population.

Characteristics	Overall (n = 213)	Low ejection fraction (n = 35)	Normal Ejection Fraction (n = 178)	P value
**Clinical**				
Age (years), [SD]	52.4 (18.3)	55.6 (18.9)	51.8 (18.2)	0.268
Male sex, n (%)	106 (49.8)	21 (19.8)	84 (79.2)	0.311
Female sex, n (%)	107 (50.2)	14 (13.1)	94 (87.9)	-
BMI (kg/m^2^), [SD]	27.1 (25.1)	25.1	27.4 (0.4)	0.194
Obesity (BMI ≥ 30), (%)	25.6	16.7	27.1	NA
Heart rate (beats/min), [SD]	77.8 (18.3)	93.4 (20.4)	74.2 (16.6)	<0.001
**Echocardiography**				
LVEDd (mm), [SD]	50.9 (8.1)	59.1 (9.3)	49.4 (6.8)	<0.001
IVSd (mm), [SD]	9 (2.4)	9.5 (2.1)	8.9 (2.3)	0.237
LVPWd (mm), [SD]	9 (2.3)	9.4 (2.2)	8.9 (2.3)	0.228
RWT, [SD]	0.36 (0.11)	0.33 (0.1)	0.37 (0.120	0.046
LV Mass (g)	169.3 (73)	228.7(88.5)	157.6(64)	<0.001
EF (Simpson), %[SD]	63.4 (16.1)	33.1 (11.4)	69.3 (8.2)	<0.001
EF (Teicholz), %[SD]	63.6 (15.7)	34.7 (12.1)	69.3 (8.3)	<0.001
FS, % [SD]	35.9 (1.1)	17.3 (6.9)	39.6 (7)	<0.001
VTI (cm), [SD]	22.7 (7.8)	14.8 (5.2)	23.9 (7.5)	<0.001
MAPSE (mm), [SD]	14.2 (3.5)	9.7 (2.7)	15.1 (3)	<0.001
ARSE (mm), [SD]	9.6 (2.7)	5.9 (2.2)	10.3 (2.2)	<0.001

SD: Standard Deviation, BMI: Body Mass Index, LV: Left Ventricle, EF: Ejection Fraction, FS: fractional Shortening, VTI: Velocity Time Integral of LV outflow tract, MAPSE: Mitral Annular Plane Systolic Excursion, ARSE: Aortic Root Systolic excursion, LVED: Left Ventricular End Diameter in diastole (d), IVSd: Interventricular septum in diastole, LVPWd: Left Ventricular Posterior Wall in diastole, RWT: Relative Wall Thickness

### Correlations of ARSE with LV systolic function indices, and diagnostic performance

**Fig 2:**
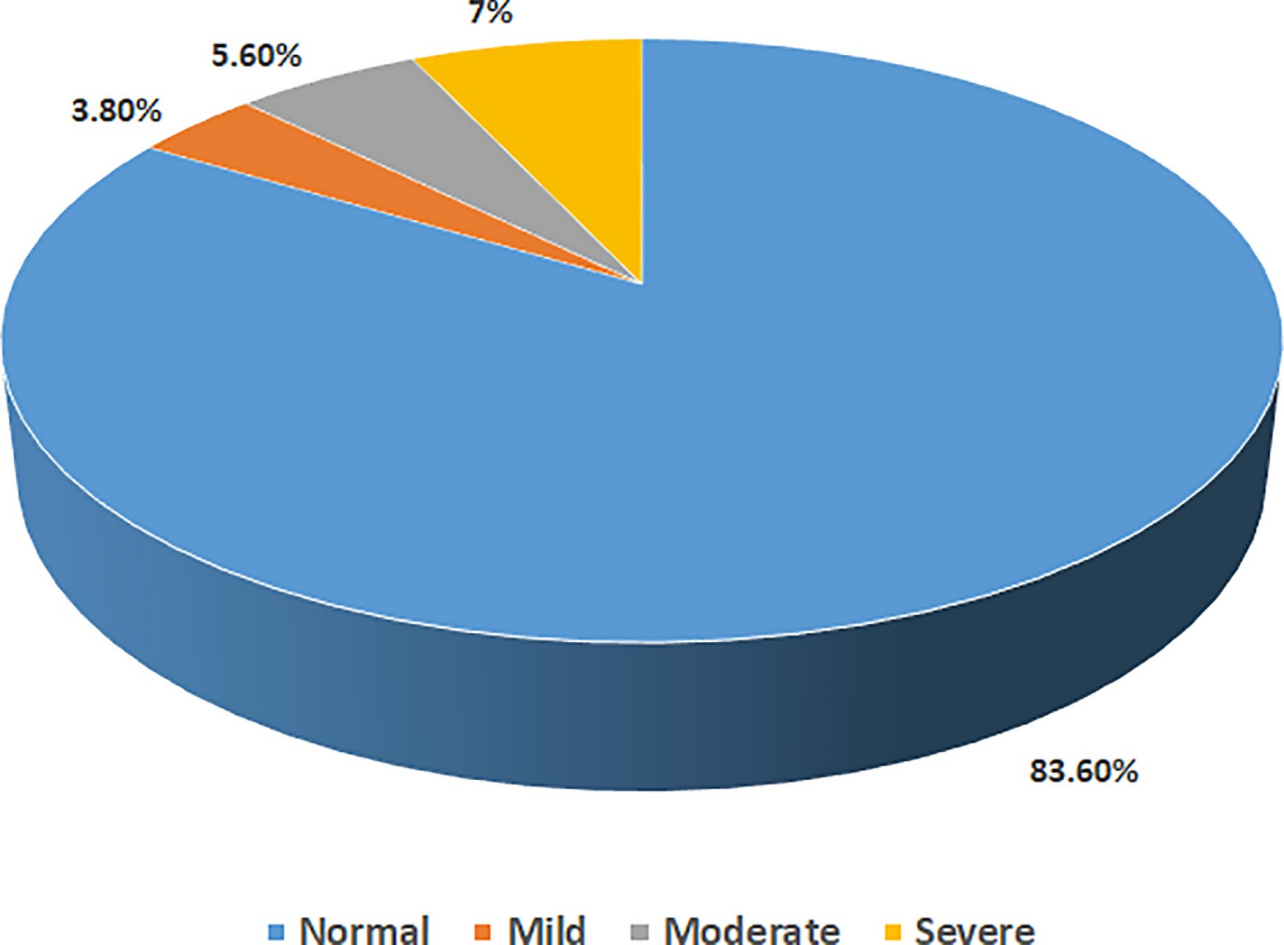
Distribution of left ventricular ejection fraction according to the severity.

The prevalence of LV systolic dysfunction varied depending on the method considered. The rate was highest when it was evaluated using Teicholz method (17.4%, [95% CI: 12.3–22.5]) or Simpson method (16.4%, [95% CI: 11.4–21.4]), lower when using Fractional Shortening (FS) method (14.1%, [95% CI: 9.4–18.8]) or VTI method (13.6%, [95% CI: 9–18.2]), and least when using MAPSE method (5.2%, [95% CI: 2.2–8.2]). The distribution of LV Ejection Fraction according to the modified Simpson method is shown in Fig 2. Normal Left Ventricular ejection fraction was seen in 83.6% of participants. Also, 56.3% had normal LV geometry, 14.1% had concentric remodeling, 10.8% had concentric LV hypertrophy, and 18.8% had eccentric LV hypertrophy.

**Fig 3:**
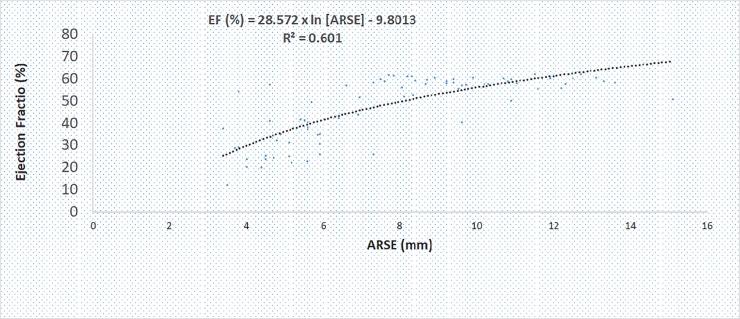
Scatter plot of ARSE (mm) versus LE ejection fraction (Simpson).

Aortic Root Systolic Excursion (ARSE) significantly correlated with the LV systolic function indices (Fig 3). This was highest for Simpson method (r = 0.619, p<0.001) and Teicholz method (r = 0.634, p<0.001), followed by FS method (r = 0.596, p<0.001) and MAPSE method (r = 0.583, p<0.001), and least for ITV method (r = 0.319, p<0.001).

**Fig 4:**
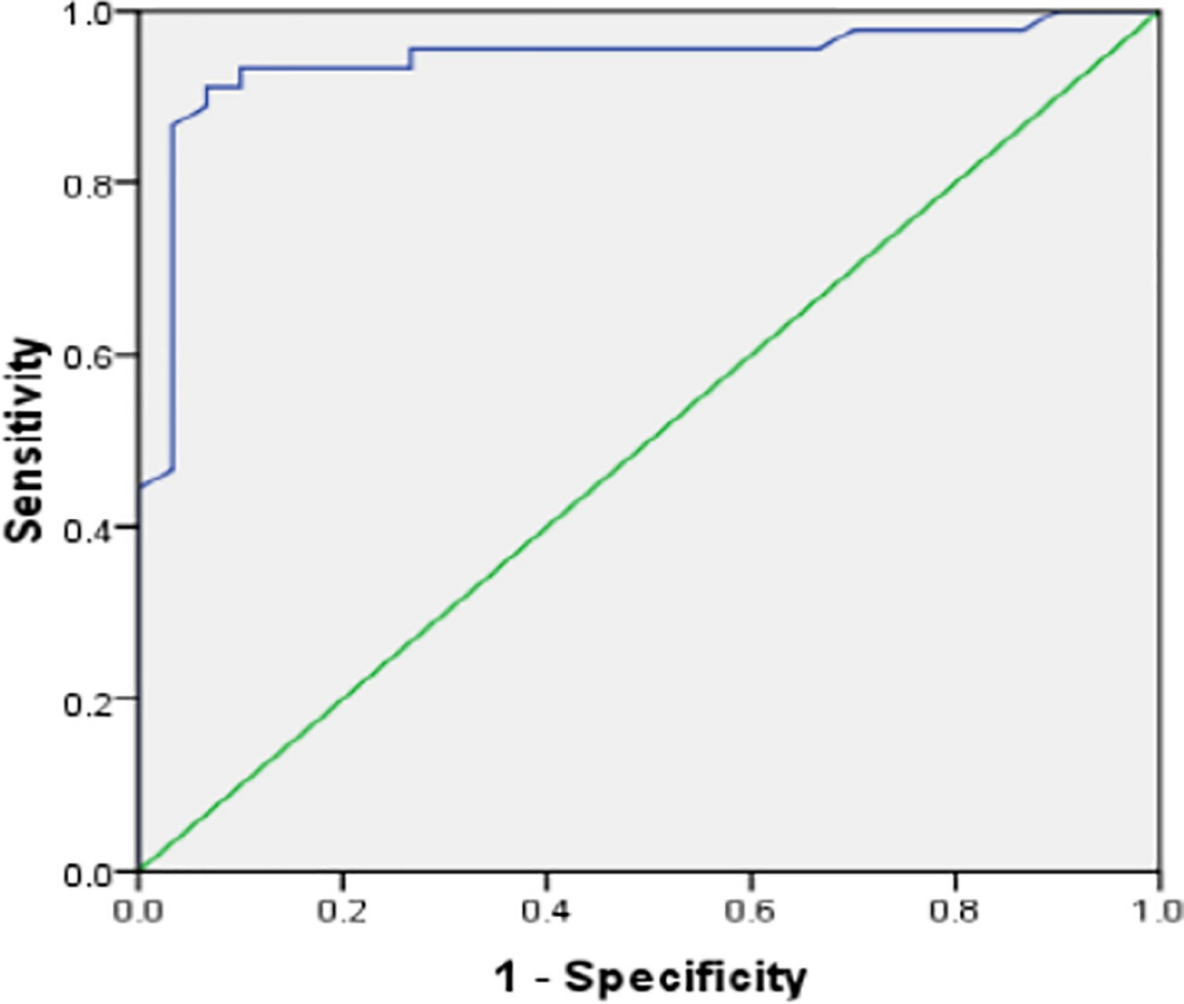
ROC Curve for optimal ARSE in detecting an ejection fraction < 55% by Simpson.

Optimal cut-offs of ARSE to detect LV systolic dysfunction derived from ROC curves (Fig 4) are shown in Table 2. This varied between 6.10 mm and 6.97 mm (Overall mean ARSE ≈ 6.60 mm).

**Table 2:** Left ventricular systolic function indices and the diagnostic performance of ARSE with optimal cut-offs from ROC curves.

Characteristics	Simpson < 55%	Teicholz < 55%	FS < 25%	VTI < 16mm	MAPSE < 10mm
Optimal Cut-Off of ARSE (mm) from ROC Curves	6.92	6.97	6.5	6.1	6.35
AUROC	0.916	0.93	0.974	0.89	0.955
Sensitivity, %	82.9	83.8	90	62.1	90.9
Specificity, %	97.2	97.7	97.8	97.1	95.3
Accuracy, %	94.8	95.3	96.7	92	94.9
Positive Predictive Value, %	85.3	88.6	87.1	78.3	66.7
Negative Predictive value, %	96.7	96.6	98.4	93.8	99.0
Positive likelihood Ratio	29.5	36.9	41.2	21.4	19.3
Negative likelihood Ratio	0.18	0.17	0.10	0.39	0.10

FS: Fractional Shortening, VTI: Velocity Time-Integral of Left Ventricular Outflow Tract, MAPSE: Mitral Annular Plane Systolic Excursion

ARSE showed an overall good performance (AUROC >90%) in detecting LV systolic dysfunction, with a sensitivity that varied between 62% and 91%, and a specificity > 95%. The Odds Ratio (OR) of having a low LVEF when ARSE ≤ 6.5 mm is 233.3 (95% CI: 57–955.9).

### Models predicting left ventricular ejection fraction by the Simpson method

Simple regression models to best predict LV ejection fraction (Simpson method) from ARSE are shown in Table 3. Of all the possible models, three appeared to compete for the data. The logarithmic model appeared best in predicting LV ejection fraction as it explained 60.2% of the data, followed by the power model that explained 56.7% of the data, and the linear model that explained 53.6% of the data. Modifying (simplifying) the logarithmic model by excluding the constant portion and rounding-up the B score to have: (LV ejection fraction (%) = 29 x In [ARSE]; where ARSE is in mm), we obtained reasonable estimates of high and low LV ejection fraction by the Simpson method compared to the other competing models.

**Table 3:** Regression equations to estimate LV ejection fraction by the Simpson method.

Model	Regression Equation	R^2^ Coefficient	p value
Logarithmic	28.572 x In [ARSE]– 9.801	0.602	<0.001
Power	10.44 x [ARSE]^0.7265^	0.567	<0.001
Linear	3.7 x [ARSE] + 18.66	0.536	<0.001

ARSE: Aortic Root Systolic Excursion in mm, R^2^: Coefficient of determination

### Bland-Altman plots of measured LVEF by Simpson method, and estimated LVEF from ARSE amplitude generic equation

**Fig 5:**
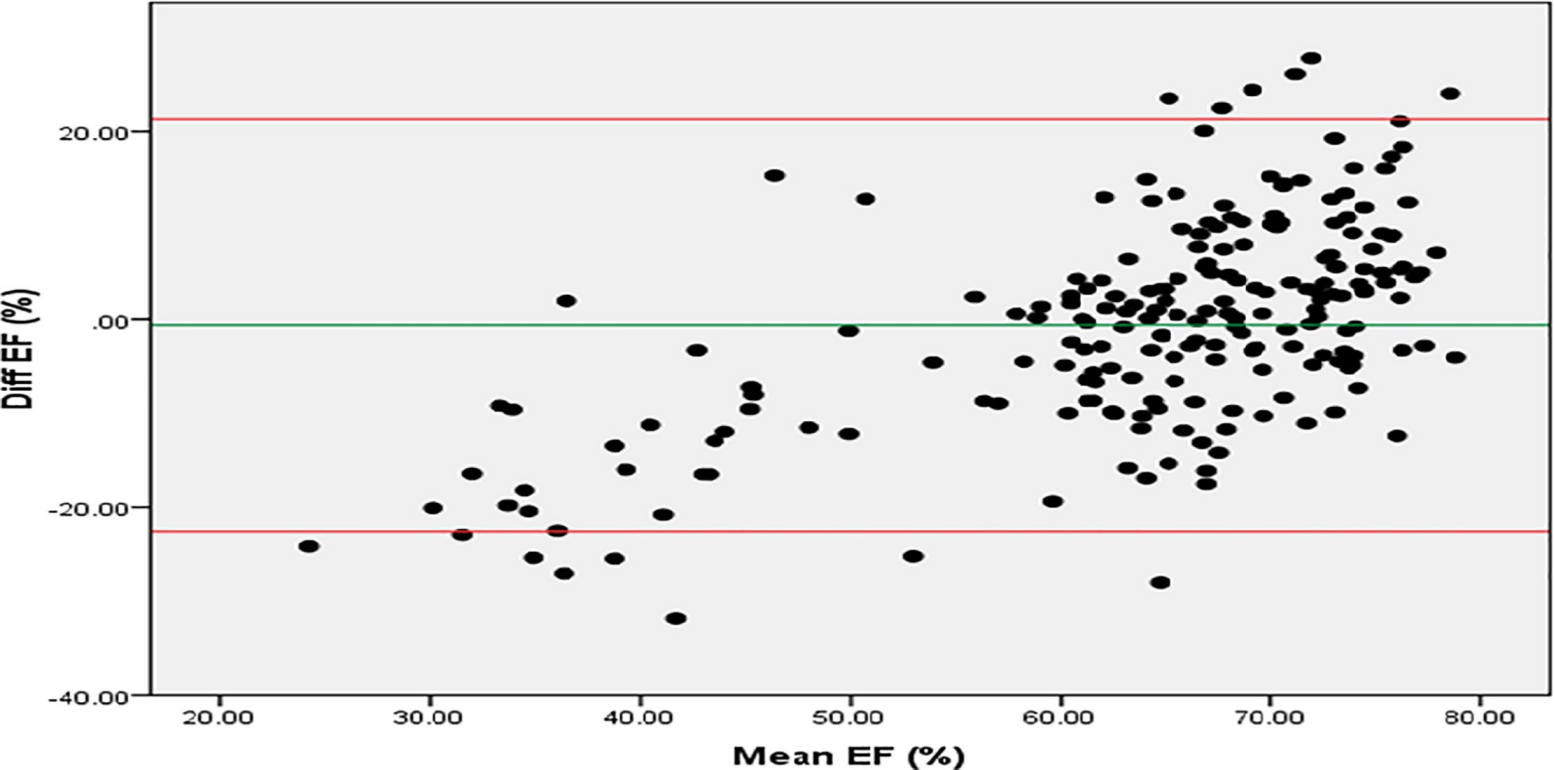
Bland-Altman plot of agreement of the measured LVEF by Simpson method, and the estimated LVEF from ARSE generic equation.

The agreement of the modified logarithmic equation in predicting Left Ventricular ejection fraction assessed using the Simpson method is shown in Fig 5. This was evaluated using the Bland-Altman plot. The mean of the difference of measured and estimated LVEF was -0.61 ± 11.2% (p = 0.425). There was a proportional bias (β coefficient: 0.579, p<0.001), that persisted even after log-transformation of the data. At high LVEF (>70%), the modified log equation significantly under-estimated the LVEF (three participants). At very low LVEF (<30%), the modified log equation significantly over-estimated the LVEF (one participant).

### External validity of ARSE in estimating LVEF

The validity of ARSE ≤ 6.5 mm in predicting a low LVEF (<55%) was validated in an independent sample of 32 echocardiograms of Caucasians, of whom 14 (43.8%) were males. Their mean age was 62 ± 15.3 years, and ranged from 29 to 87 years. Low LVEF was seen in 8 (25%), and ARSE ≤ 6.5 mm was seen in 10 (31.3%). ARSE ≤ 6.5 mm predicted a low LVEF with a sensitivity of 87.5% (95% CI: 47.4–99.7), specificity of 87.5% (95% C: 67.4–97.3), positive predictive value of 70% (95% CI: 44–87.4), negative predictive value of 95.5% (95% CI: 76.9–99.3), positive likelihood ratio of 7 (95% CI: 2.4–20), and negative likelihood ratio of 0.14 (95% CI: 0.02–0.9). The accuracy was 87.5% (95% CI: 71–96.5), and the odds (OR) of having a low LVEF when ARSE ≤ 6.5 mm in this group of people was 49 (95% CI: 4.4–551).

## Discussion

In this prospectively conducted cross-sectional and analytic study of 213 echocardiograms on the correlations of Left Ventricular (LV) functions with Aortic Root Motion in Systole and Diastole, 1: We have shown that Aortic Root Systolic Excursion (ARSE) correlates moderately with LV systolic function indices with a good sensitivity, specificity, accuracy, predictive values, and likelihood ratios. 2: We have established that LV systolic function assessed by the modified Simpson method can best be estimated from ARSE using the modified generic equation: LVEF (%) = 29 x In [ARSE], where ARSE is in millimeters (mm). An ARSE ≤ 6.5 mm suggests an LVEF < 55% measured using the Simpson method.

The idea of studying movements of parts of the heart such as the aorta as a surrogate of heart function should probably be age long, as M-mode was the first modality to be described [8]. Few studies have quantified these movements with the different modalities of evaluating LV systolic functions in the new era. The good correlations of LV systolic functions with ARSE has been reported by Ünlüer et al [11] and Burggraf et al [15]. However, significant differences with our study are worth noting. We included a larger cohort of participants, and measurements were carried-out by an experienced Cardiologist, as opposed to that of Ünlüer et al [11], where data were partly collected by emergency physicians and an experienced Cardiologist. With their generic equation: LVEF = 20 + 40 x ARSE (cm), and the set threshold of 6.0 mm, the estimated LVEF is 46%, far lower than the recommendations of ASE [9]. Also, their generic equation failed to detect ejection fraction less than 20%. We developed three generic equations and noticed that the linear model explained fewer data points. Our modified generic equation: LVEF = 29 x In [ARSE] performed better. For a set threshold of 6.6 mm, the estimated EF is ≈ 55%. We could estimate LVEF lower than 20%. For example, for an ASRE of 1.5 mm the LVEF is ≈ 12%. Using the examples put forth by Ünlüer et al [11] on our generic equation, an ARSE of 3.8 mm gives an LVEF of 38.7% versus 38%. An ARSE of 12.1 mm gives an LVEF of 72.3% versus 70%. Our work seems to be an improvement of that reported by Ünlüer et al [11] in terms of optimal threshold and the model fit. We tested three models (Table 3) to best explain the data. Our linear model 18.66 + 37x ARSE (cm) was comparable to that reported by Ünlüer et al. 20 + 40 x ARSE (cm). This linear model explained 53.6% of the data. The best model we are proposing is the simplified Logarithmic model 29 x In ARSE (mm) which explained 60.2% of the data. However, the model by Ünlüer et al can be computed off-hand, while our model needs a calculator.

Another surrogate of LV systolic function that is easy to measure by the less skilled and busy physician is the E-point septal separation (EPSS) [16,17]. However, this did not reliably predict fractional shortening [18]. The EPSS has an un-acceptable low specificity of 51.6% at the 7.0 mm set threshold. We argue that the EPSS reflects more of LV chamber size as it is measured in diastole. With a large LV diameter in diastole as in athletes, we expect an increase in the EPSS without necessarily having low LV ejection fraction. Also, in the case of an infiltrative LV hypertrophy with hypocontractility, the EPSS will not necessarily be greater than 7.0 mm. This makes ARSE a more reliable estimate of LV systolic functions than the EPSS for the rapid evaluation of LV systolic dysfunction.

ARSE in low-income settings like ours has some clinical utilities. There are about 64 cardiologists in Cameroon, for a total population of about 23 million inhabitants. Most of them are concentrated in two major neighboring cities. Thus, most patients with cardiovascular disorders are cared for by general practitioners (GPs) who lack specialized skills in cardiac ultrasound. There is also an increasing number of low-technology echographs in our setting that lack the sophisticated cardiac algorithms. There is clearly the need for some task shifting to GPs, including basic cardiac ultrasound techniques with ARSE evaluation. Monitoring and guidance of GPs in distant areas by cardiologists can be carried out through telecommunication which is widely available in our setting. In low-income settings, GPs can be trained to assess ARSE, and abnormal ARSE (≤6.5mm) will warrant referral to the nearest cardiologist for a formal echocardiography. However, the reproducibility of ARSE measurements by GPs is not known. There is a need for a well-designed study to assess the reproducibility of ARSE measurements as well as other LV systolic function indices by GPs. Even in busy cardiac ultrasound laboratories in low income, ARSE can be used to give an idea of the expected LV ejection fraction. Further studies are needed to see if ARSE gives information beyond the LV ejection fraction–such as the aortic root rigidity.

Despite this improvement, we encountered some difficulties in measuring ARSE in certain participants. Some participants presented with increased or multiple layer echogenicity of the posterior or far aortic wall, making precise measurements of ARSE difficult. Such cases occurred with breathing. These were considered technically difficult and the measurements uncertain. Such data were not included in the analysis. Also, some participants appeared to have asymmetry in the movements of the aortic wall, with the anterior or near wall having a higher amplitude of movements than the posterior portion despite perpendicular sectioning in M-mode. All measurements were performed on the posterior wall of the aortic root, so as not to violate the protocol. The effect of sampling ARSE from the anterior wall in such conditions remains uncertain.

Our findings should be considered in the light of some limitations. No intra-observer or inter-observer variability testing was carried-out. In order to ensure measurements as per protocol, the measurements were carried out in the presence of the principal investigator (AMJ) who worked with the cardiologist (BH). At least three measurements per parameter were obtained when the first two measurements were markedly different. More precise echocardiographic measurements of LV volumes aiding with contrast (SonoVue) were not used. 3D echocardiographic measurements of LV volume that correlate well with MRI measurements were also not used. This technique cannot be generalized to all patients as we excluded patients < 18 years, patients with significant LV outlet obstruction, large pericardial effusion, and markedly enlarged left atrium. However, about ¾ of cases of heart failure in low-income settings are accounted for by hypertensive heart disease, rheumatic heart disease, and the dilated cardiomyopathies [2]. Despite these limitations, this study has some merits. The sample size was large enough, about seven times the required number of participants. This makes our work more representative, and our findings more reliable. This high sample size was obtained due to the high volume of cardiac ultrasound performed by the echocardiography laboratory, and no objection on the part of the participants on the use of their data. Our findings were also validated against a small group of older patients, with a different racial background. The small number of this test group might reduce the statistical precisions. Also, the older age of this group of patients might result in a lower test performance due to aortic rigidity. Further validation of our findings in a larger and younger cohort is needed, with the determinants of aortic rigidity taken into account.

## Conclusions

Aortic Root Systolic Excursion (ARSE) is an acceptable surrogate of LV systolic functions at set threshold of < 6.6 mm (approximately ≤ 6.5 mm) in predicting an LVEF < 55% according to the Simpson method. In this prospectively conducted cross-sectional study on the correlations of Left Ventricular (LV) functions with ARSE, we have confirmed that ARSE correlates moderately with LV systolic functions with a good sensitivity, specificity, accuracy, predictive values, and likelihood ratios. We have also established that LV systolic function assessed by the modified Simpson method can best be estimated from ARSE using the modified generic equation: LVEF (%) = 29 x In [ARSE), where ARSE is in millimeters (mm). Despite the improvements shown in our study, we recommend further research on Aortic Root Systolic Excursion (ARSE) so as to validate our findings. In practice, GPs in low income settings can be trained to measure ARSE, and an abnormal ARSE will warrant referral to a cardiologist for formal echocardiography.

## Supporting information

S1 Dataset Aortic Root Systolic Excursion study data. (XLS)

S1 Fig How to sample. ARSE is measured at the far wall of the aortic root using the TM mode. ARSE is measure between the yellow lines (red arrow).
(TIF)
