# Supplementary figures and images for: Correlations of left ventricular systolic function indices with aortic root systolic excursion (ARSE): A cross-sectional echocardiographic study
Source: PLoS One. 2018 Nov 6;13(11):e0206199. doi: 10.1371/journal.pone.0206199 (PMC6219773; doi:10.1371/journal.pone.0206199)

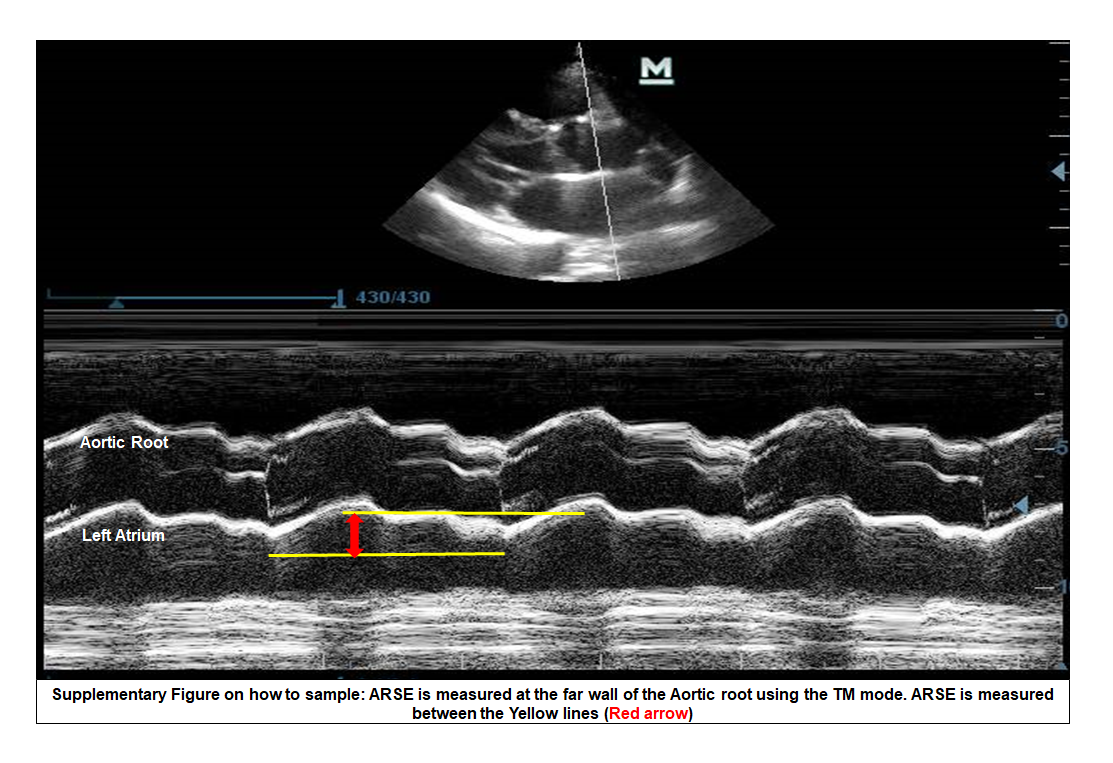

Supplement: S1 Fig — ARSE is measured at the far wall of the aortic root using the TM mode. ARSE is measure between the yellow lines (red arrow). (TIF) [file pone.0206199.s002.tif]
